# The Dual PI3K/mTOR Inhibitor NVP-BEZ235 Induces Tumor Regression in a Genetically Engineered Mouse Model of *PIK3CA* Wild-Type Colorectal Cancer

**DOI:** 10.1371/journal.pone.0025132

**Published:** 2011-09-26

**Authors:** Jatin Roper, Michael P. Richardson, Wei Vivian Wang, Larissa Georgeon Richard, Wei Chen, Erin M. Coffee, Mark J. Sinnamon, Lydia Lee, Peng-Chieh Chen, Roderick T. Bronson, Eric S. Martin, Kenneth E. Hung

**Affiliations:** 1 Division of Gastroenterology, Department of Medicine, Tufts Medical Center, Boston, Massachusetts, United States of America; 2 Department of Genetics, Brigham and Women's Hospital, Harvard Medical School, Boston, Massachusetts, United States of America; 3 Dana Farber/Harvard Cancer Center, Harvard Medical School, Boston, Massachusetts, United States of America; University of Barcelona, Spain

## Abstract

**Purpose:**

To examine the *in vitro* and *in vivo* efficacy of the dual PI3K/mTOR inhibitor NVP-BEZ235 in treatment of *PIK3CA* wild-type colorectal cancer (CRC).

**Experimental Design:**

*PIK3CA* mutant and wild-type human CRC cell lines were treated *in vitro* with NVP-BEZ235, and the resulting effects on proliferation, apoptosis, and signaling were assessed. Colonic tumors from a genetically engineered mouse (GEM) model for sporadic wild-type *PIK3CA* CRC were treated *in vivo* with NVP-BEZ235. The resulting effects on macroscopic tumor growth/regression, proliferation, apoptosis, angiogenesis, and signaling were examined.

**Results:**

*In vitro* treatment of CRC cell lines with NVP-BEZ235 resulted in transient PI3K blockade, sustained decreases in mTORC1/mTORC2 signaling, and a corresponding decrease in cell viability (median IC_50_ = 9.0–14.3 nM). Similar effects were seen in paired isogenic CRC cell lines that differed only in the presence or absence of an activating *PIK3CA* mutant allele. *In vivo* treatment of colonic tumor-bearing mice with NVP-BEZ235 resulted in transient PI3K inhibition and sustained blockade of mTORC1/mTORC2 signaling. Longitudinal tumor surveillance by optical colonoscopy demonstrated a 97% increase in tumor size in control mice (p = 0.01) vs. a 43% decrease (p = 0.008) in treated mice. *Ex vivo* analysis of the NVP-BEZ235-treated tumors demonstrated a 56% decrease in proliferation (p = 0.003), no effects on apoptosis, and a 75% reduction in angiogenesis (p = 0.013).

**Conclusions:**

These studies provide the preclinical rationale for studies examining the efficacy of the dual PI3K/mTOR inhibitor NVP-BEZ235 in treatment of *PIK3CA* wild-type CRC.

## Introduction

In 2011, colorectal cancer (CRC) will continue to be the third most common cause of cancer-related mortality in the U.S [1]. Despite the growing arsenal of chemotherapeutic agents, the median survival for patients with metastatic CRC is still less than 20 months, which underscores the urgent need for the development of novel therapeutic approaches [2].

Mammalian target of rapamycin (mTOR) is a serine/threonine kinase that regulates cellular proliferation and apoptosis. mTOR binds regulatory associated protein of mTOR (Raptor) and mammalian LST8/G-protein β-subunit like protein (mLST8/GβL) to form the mTOR complex 1 (mTORC1), which promotes translation through phosphorylation of p70 S6 kinase (S6K), S6 ribosomal protein (S6), and eukaryotic initiation factor 4E binding protein 1(4E-BP1). Alternatively, mTOR can bind rapamycin-insensitive companion of mTOR (Rictor), mLST8/GβL, and mammalian stress-activated protein kinase interacting protein 1 (mSIN1) to form mTOR complex 2 (mTORC2) [3], [4].

The upstream phosphatidylinositol 3-kinase (PI3K) signaling pathway can activate mTOR. Class IA PI3Ks are activated by growth factor receptor tyrosine kinases (RTKs) and are composed of a heterodimer consisting of a p110α/p110β catalytic and a p85 regulatory subunit [5]. The *PIK3CA* (phosphatidylinositol 3-kinase, catalytic, α-polypeptide) gene that encodes p110α is frequently mutated in many human cancers, including CRC [6]. Point mutations in *PIK3CA* cluster at two hotspots: E545K in the helical domain (exon 9) and H1047R in the catalytic kinase domain (exon 20). These mutations increase p110α activity and promote CRC cell growth, invasion, and migration *in vitro* via activation of the PI3K pathway [7]. Mutations in the helical and catalytic domains of *PIK3CA* confer essentially identical phenotypes in human CRC cell lines [7]. AKT is a critical downstream effector of the PI3K pathway and promotes cell growth and survival via a number of mechanisms, including phosphorylation of TSC2, which results in mTORC1 activation [5]. Full activation of AKT is achieved after phosphorylation at Thr308 and Ser473 by PDK1 and mTORC2, respectively [5], [8]–[11].

Because of its central role in carcinogenesis, mTORC1 blockade is an attractive therapeutic strategy for CRC. Treatment of Apc Δ716 mice with the mTORC1 inhibitor everolimus inhibits cellular proliferation and tumor angiogenesis, resulting in a decrease in both number and size of intestinal tumors [12]. We have recently reported that treatment of a genetically engineered mouse (GEM) model for sporadic CRC with the mTORC1 inhibitor rapamycin results in an 80% reduction in individual tumor growth, as observed by longitudinal colonoscopy surveillance [11]. However, the clinical efficacy of mTORC1 blockade may be attenuated by the concomitant loss of an mTORC1-dependent negative feedback loop on PI3K signaling (reflected by increased AKT phosphorylation at Thr308), and continued mTORC2-mediated activation of AKT through phosphorylation at Ser473 [9]–[14]. Indeed, a Phase I clinical trial examining the efficacy of the mTORC1 inhibitor everolimus in advanced solid tumors demonstrated modest benefit in only one of 16 colorectal cancer patients and overall increased phosphorylation of AKT at Ser473 [13]. Taken together, it appears that therapeutic strategies in which PI3K and mTOR are concurrently inhibited may be most efficacious.

NVP-BEZ235 (Novartis) is a dual pan-class I PI3K and mTOR kinase inhibitor that has been demonstrated to reduce tumor growth in a number of different xenograft and several genetically engineered mouse (GEM) models and is currently in clinical trials [14]–[50]. There has been suggestion that use of such agents may be limited to tumors with activating mutations in *PIK3CA* [51], [52]. As activating *PIK3CA* mutations are seen in only 17% of CRC, this would imply such agents may be targeted towards only a small proportion of patients [53]. Because NVP-BEZ235 inhibits the wild-type and mutant forms of *PIK3CA* with comparable efficacy [32], we hypothesized that NVP-BEZ235 may have significant efficacy in the treatment of *PIK3CA* wild-type CRC.

In this manuscript, we describe results from *in vitro* treatment studies demonstrating comparable efficacy of NVP-BEZ235 against both *PIK3CA* mutant and wild-type human CRC cell lines. We also describe results from *in vivo* treatment studies demonstrating significant efficacy in a GEM model for sporadic wild-type *PIK3CA* CRC. Taken together, our findings provide a compelling preclinical rationale for clinical trials to examine the use of NVP-BEZ235 in treatment of *PIK3CA* wild-type CRC patients.

## Materials and Methods

### *In vitro* treatment of human CRC cell lines

HCT116 (*PIK3CA* mutant; kinase domain at H1047R), DLD-1 (*PIK3CA* mutant; helical domain at E545K), and SW480 (*PIK3CA* wild-type) human CRC cell lines (ATCC) and isogenic DLD-1 *PIK3CA* mutant and wild-type cells (obtained from B. Vogelstein) were maintained in DMEM (Invitrogen) with 10% FBS and 1× Penicillin/Streptomycin (Invitrogen). Cells were plated at different initial densities (HCT116: 3,000 cells/well, DLD-1: 5,500 cells/well, SW480: 4,500 cells/well, DLD-1 *PIK3CA* mutant: 7,000 cells/well, and DLD-1 *PIK3CA* wild-type: 9,000 cells/well) to account for differential growth kinetics. After 16 hours, cells were incubated with increasing concentrations of NVP-BEZ235 (Novartis), and drug-containing growth medium was changed every 24 hours. Cell viability was assessed 16 hours after the initial plating and 48 hours after initiation of drug treatment using the colorimetric MTS assay CellTiter 96® AQueous One Solution Cell Proliferation Assay (Promega), as per the manufacturer's instructions. Cell viability after drug treatment was normalized to that of untreated cells also grown for 48 hours. IC_50_ values were calculated using 4 parameter nonlinear regression in GraphPad Prism 5 (GraphPad Software). For western blot analysis, cells were plated with 0 nM or maximal inhibitory dose (500 nM) NVP-BEZ235 for 2, 6, 24, or 48 hours.

### Sequencing of colonic tumors from a GEM model for sporadic CRC

C57BL/6J *Apc* conditional knockout mice (Apc CKO) were treated with Adeno-Cre, as previously described [11]. Following necropsy, 10 tumor specimens were collected in 1 ml RNA Later (Invitrogen, Inc), stored overnight in 4°C, then removed from RNA Later and archived in −80°C. RNA was extracted from specimens using RNeasy (Qiagen, Inc.), and cDNA was generated with reverse transcriptase (Omniscript RT, Qiagen, Inc). Primers designed by the study authors were used to create 553 bp and 477 bp amplicons (PCR performed using Platinum PCR SuperMix High Fidelity, Invitrogen, Inc) spanning codons 532–554 of exon 9 (helical domain; 9F: 5′ GCAGTGTGGTGAAGTTTCCA 3′, 9R: 5′ TGGCCAATCCTTTGATTTGT 3′) and c1011–1062 of exon 20 (kinase domain; 20F: 5′ ACTGCGTGGCAACCTTTATC 3′, 20R: 5′ TGATGGTGTGGAAGATCCAA 3′) of the *Pik3ca* gene, respectively, which include mutation hotspot regions. Sanger sequencing of the amplicons was performed at the BioPolymers Facility at Harvard Medical School, and results analyzed with Sequencher 4.10.1 (Gene Codes, Inc).

### *In vivo* treatment of a GEM model for sporadic CRC

Apc CKO mice were treated with Adeno-Cre and followed by optical colonoscopy, as previously described [11]. As a colonoscopic metric for tumor size, the Tumor Size Index (TSI) was calculated as (tumor area/colonic lumen area)×100 (%). Tumor-bearing mice were randomly assigned to treatment with either control vehicle alone (n = 8) or 45 mg/kg body weight NVP-BEZ235 in 10% 1-methyl-2-pyrrolidone/90% PEG 300 (n = 8) by daily oral gavage for 28 days. The treatment dose was chosen based on literature indicating that 40–50 mg/kg body weight NVP-BEZ235 effectively treats murine tumor models without adverse effects [15], [24], [26], [28], [32], [47]. Based on pharmacokinetic studies demonstrating maximal tissue concentration one hour after NVP-BEZ235 administration, tumor-bearing mice were sacrificed one hour after final treatment dose [32]. Colonic tumor volume was assessed using calipers (width×length×height) and tumors were harvested for both western blot analysis and immunohistochemistry.

### Western blot analysis

Concentrations of whole cell or tumor lysates were determined by Bio-Rad Protein Assay (Bio-Rad). 10 µg and 25 µg protein lysate for whole cell and tumor, respectively, was separated on 10% SDS/PAGE gel, transferred to nitrocellulose membrane, blocked in 1% BSA for one hour, incubated at room temperature for two hours with primary antibody and one hour with secondary antibody. Detection was performed using the Amersham™ ECL™ Western Blot Detection Reagents (GE Healthcare). p-AKT Thr308 (1∶1000 dilution), p-AKT Ser473 (1∶2000 dilution), total AKT (1∶1000 dilution), p-S6 Ser240/244 (1∶3000 dilution), p-S6 Ser235/236 (1∶1000 dilution), S6 (1∶1000 dilution), cleaved caspase 3 (1∶1000 dilution), and cleaved PARP (1∶1000 dilution) were obtained from Cell Signaling Technologies (Beverly, MA). β-actin (1∶5000 dilution) was obtained from Santa Cruz Biotechnology (Santa Cruz, CA). Peroxidase AffiniPure Donkey Anti- Rabbit Ig secondary antibody (1∶10,000 dilution) was obtained from Jackson ImmunoResearch (West Grove, PA) [11].

### Immunohistochemistry

Five µm paraffin-embedded tissue sections were deparaffinized in xylene followed by alcohol rehydration. Antigen retrieval was performed in 1× citrate buffer (pH 6.0) (Zymed) using a Medical Decloaking Chamber (Biocare Medical). Slides were blocked at room temperature with Peroxidase Blocking Reagent (DAKO), normal donkey/rabbit serum, and Avidin/Biotin Blocking Kit (Vector Laboratories). Slides were incubated overnight at 4°C with primary antibody and 30 minutes at room temperature with secondary antibody. The Vectastain ABC kit (Vector Laboratories) was used for detection per manufacturer's instructions. Slides were stained with the Liquid DAB+Substrate Chromogen System (Dako) per manufacturer's instructions and counterstained with Mayer's hematoxylin solution and Scott's Bluing solution. p-AKT Ser473 (1∶50 dilution), p-S6 Ser240/244 (1∶50 dilution), p-S6 Ser235/236 (1∶100 dilution), were obtained from Cell Signaling Technologies (Beverly, MA). CD31 (1∶100 dilution) was obtained from Santa Cruz Biotechnology (Santa Cruz, CA). KI-67 (1∶100 dilution) was obtained from US Biological (Swampscott, MA). TUNEL assay (Apoptag) was purchased from Millipore (Billerica, MA) [11]. The KI-67 proliferation index was calculated as the mean number of KI-67 positive cells/total number of glandular cells per high power field (mean of 16 high power fields)×100, and microvessel density (MVD) was calculated as number of CD31 positive cells per high power field (mean of 16 high power fields). TUNEL positivity index was calculated as mean number of TUNEL positive cells/total number of glandular cells per high power field (mean of 16 high power fields)×100. Measurements were performed by three blinded, independent observers in four control and four treated tumors.

### Statistics

Comparisons of final tumor volume, KI-67, MVD, and apoptotic cells between control and NVP-BEZ235-treated cohorts were calculated using the two-tailed Independent-Samples T Test. Pre- and post-treatment TSI values were compared with the Wilcoxon signed-rank test. P<0.05 was considered significant for all analyses. All analyses were calculated using SPSS 18.0 for Windows (IBM, Inc).

## Results

### *In vitro* NVP-BEZ235 treatment of human CRC cell lines decreases cellular proliferation but has no effect on apoptosis

**Figure 1:**
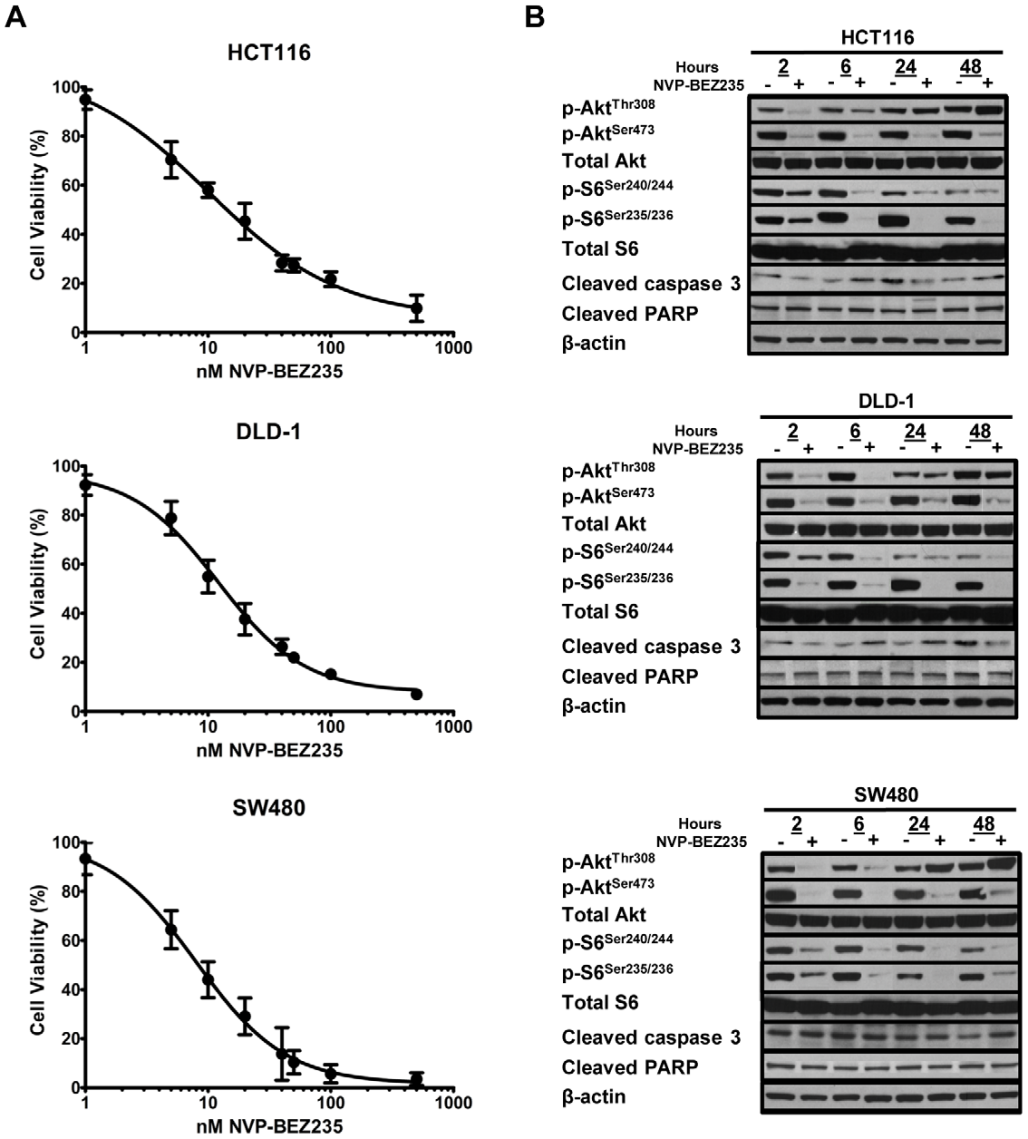
NVP-BEZ235 treatment results in decreased cell viability, transient PI3K blockade inhibition, and sustained mTORC1/mTORC2 blockade. (A) Cell viability of HCT116, DLD-1, and SW480 CRC cell lines was assessed by MTS assay after treatment with increasing concentrations (0–500 nM) of NVP-BEZ235 for 48 hours. Results shown are the mean of three independent experiments. (B) Western blot analysis for p-AKT^Thr308^, p-AKT^Ser473^, p-S6^Ser240/244^, p-S6^Ser235/236^, cleaved caspase 3, and cleaved PARP was performed after 2, 6, 24, and 48 hours incubation with (−) 0 or (+) 500 nM NVP-BEZ235.

To examine the effects of *in vitro* NVP-BEZ235 treatment on cellular viability, three human CRC cell lines (HCT116, DLD-1, and SW480) were treated with increasing amounts of NVP-BEZ235 for 48 hours, and cellular viability was assessed by a colorimetric MTS assay. These studies revealed a similar dose-dependent decrease in viability after NVP-BEZ235 treatment (mean IC_50_ of three separate experiments = 14.3±6.4, 9.0±1.5, and 12.0±1.6 nM for HCT116, DLD-1, and SW480 cell lines, respectively; p = 0.74) for all three cell lines (Figure 1A). To determine if the observed decrease in cellular viability after NVP-BEZ235 treatment resulted from an induction of apoptosis, western blot analysis for cleaved caspase-3 and cleaved PARP was performed, revealing no increase in these apoptotic markers with NVP-BEZ235 treatment (Figure 1B). Taken together, these studies suggest that *in vitro* treatment with NVP-BEZ235 results in an equivalent decrease in cellular proliferation in two CRC cell lines harboring distinct *PIK3CA* mutations (HCT116 and DLD-1) as well as in a *PIK3CA* wild-type colorectal cancer cell (SW480), with no effect on apoptosis.

### *In vitro* NVP-BEZ235 treatment of human CRC cell lines results in sustained mTORC1 and mTORC2 inhibition, but transient PI3K blockade

To examine the effects of *in vitro* NVP-BEZ235 treatment on PI3K (p-AKT^Thr308^), mTORC1 (p-S6^Ser235/236^ and p-S6^Ser240/244^), and mTORC2 (p-AKT^Ser473^) mTOR signaling, western blot analysis was performed. After two hours of NVP-BEZ235 treatment, levels of p-AKT^Thr308^, p-AKT^Ser473^, p-S6^Ser235/236^, and p-S6^Ser240/244^ were all significantly decreased. Whereas a sustained decrease was observed in the levels of p-AKT^Ser473^, p-S6^Ser235/236^, and p-S6^Ser240/244^, full inhibition of p-AKT^Thr308^ was lost in as little as six hours (Figure 1B). Taken together, these results suggest that *in vitro* NVP-BEZ235 treatment results in sustained inhibition of mTORC1 and mTORC2 signaling, but that PI3K blockade is transient.

### The efficacy of *in vitro* NVP-BEZ235 treatment of human CRC cell lines does not depend on PIK3CA mutational status

**Figure 2:**
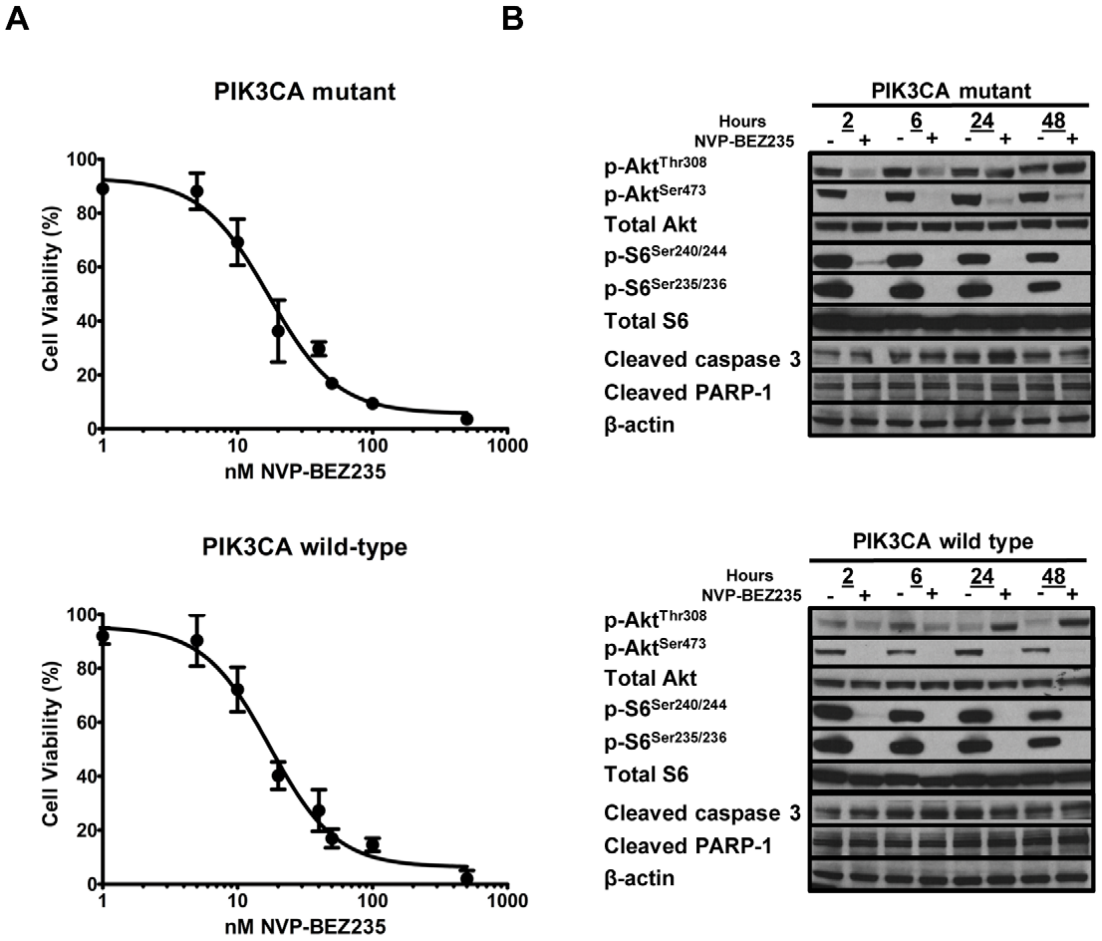
Effects of NVP-BEZ235 on cell viability and PI3K/mTOR signaling are independent of *PIK3CA* status. (A) Cell viability of mutant and wild-type isogenic *PIK3CA* cells was assessed by MTS assay after treatment with increasing concentrations (0–500 nM) of NVP-BEZ235 for 48 hours. Results shown are the mean of four independent experiments. (B) Western blot analysis for p-AKT^Thr308^, p-AKT^Ser473^, p-S6^Ser240/244^, p-S6^Ser235/236^, cleaved caspase 3, and cleaved PARP was performed after 2, 6, 24, and 48 hours incubation with (−) 0 or (+) 500 nM NVP-BEZ235.

The comparable efficacy of NVP-BEZ235 treatment in *PIK3CA* mutant (HCT116 and DLD-1) and *PIK3CA* wild type (SW480) human CRC cell lines suggests that its clinical efficacy might not be limited to those patients whose tumors contain activating *PIK3CA* mutations. To further examine this possibility, we assessed the effect of NVP-BEZ235 on cellular proliferation and intracellular cell signaling in paired *PIK3CA* mutant and wild-type cell lines. The *PIK3CA* mutant cell line was derived from DLD-1 through disruption of the wild-type *PIK3CA* allele by targeted homologous recombination, whereas the *PIK3CA* wild-type cell line was derived through disruption of the mutant *PIK3CA* allele (a kind gift from B. Vogelstein) [7]. As such, the genetic composition of these cells differs only at the *PIK3CA* locus, making these otherwise perfect isogenic controls. We found similar IC50's for NVP-BEZ235 in both *PIK3CA* mutant and wild-type DLD-1 cells (mean IC_50_ of three separate experiments = 15.1±6.0 and 12.1±4.3 nM for DLD-1 *PIK3CA* mutant and wild-type cell lines, respectively; p = 0.82, Figure 2A). Western blot analysis of p-AKT and p-S6 revealed sustained inhibition of p-AKT^Ser473^, p-S6^Ser235/236^, and p-S6^Ser240/244^; however, the inhibition of p-AKT^Thr308^ was transient in both cell lines (Figure 2B). Furthermore, NVP-BEZ235 treatment did not increase levels of cleaved caspase-3 and cleaved PARP in either cell line (Figure 2B). Overall, these findings suggest that *PIK3CA* mutational status does not predict the efficacy of NVP-BEZ235 treatment in human CRC cell lines.

### *In vivo* NVP-BEZ235 treatment induces tumor regression in a GEM model for sporadic PIK3CA wild-type CRC

**Figure 3:**
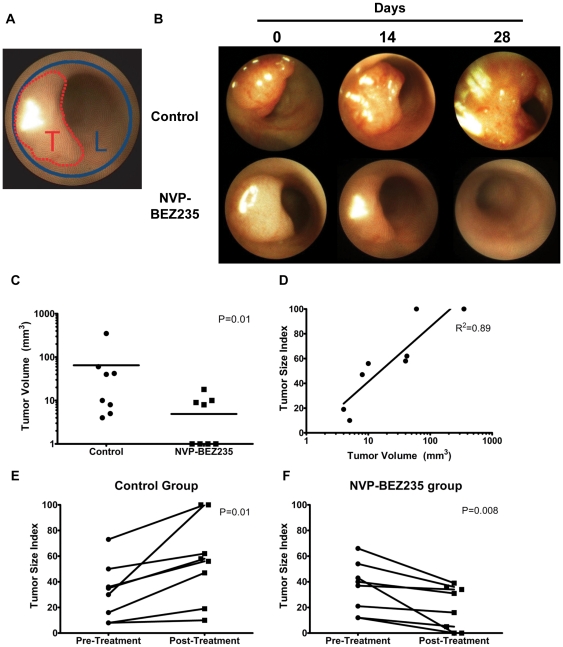
*In vivo* treatment of a GEM model for sporadic CRC results in tumor regression. Mice with colonic tumors were randomized to treatment with control diluent (N = 8) or 45 mg/kg NVP-BEZ235 (N = 8) by daily oral gavage for 28 days. Resulting colonic tumor growth or regression was serially examined by optical colonoscopy. (A) Tumor Size Index (TSI) was calculated as Tumor Area (T) divided by Lumen Area (L) ×100. (B) Representative tumor colonoscopy still images during the 28 day treatment period. (C) Tumor volume of control and NVP-BEZ235-treated tumors at necropsy (mean 65 mm^3^ vs. 5 mm^3^; p = 0.01). (D) Final TSI vs. Tumor Volume (R^2^ = 0.89, P<0.0001). Change in mean TSI for (E) control (32% pre-treatment vs. 57% post-treatment, P = 0.01) and (F) treated (32% vs. 20%, P = 0.02) cohorts.

To examine the effects of *in vivo* NVP-BEZ235 treatment on colonic tumor growth, we used a novel GEM model for sporadic CRC that we have recently described [11]. Adenovirus expressing Cre recombinase (Adeno-Cre) was used to induce colonic tumors in floxed *Apc* mice. Tumors from 10 mice were analyzed for the presence of activating mutations by direct sequencing of exons 9 and 20 of the *Pik3ca* gene. No mutations were identified, which suggests that these mice are representative of *PIK3CA* wild-type CRC. Optical colonoscopy was used to randomize treatment of comparably sized colonic tumors with control drug vehicle or 45 mg/kg NVP-BEZ235 by daily oral gavage for 28 days. We noted no toxicity or side effects during this drug treatment regimen. Subsequent longitudinal growth or regression of individual colonic tumors was determined by optical colonoscopy, as previously described [11]. For each tumor, a relative Tumor Size Index (TSI) metric was calculated as tumor size (T) normalized to colonic luminal area (L) (Figure 3A).

A representative time course of colonoscopy images for control (n = 8) and NVP-BEZ235-treated tumors (n = 8) is shown in Figure 3B. Tumor volume at necropsy was significantly larger in control vs. treated groups (65 mm^3^ vs. 5 mm^3^; p = 0.01; Figure 3C). In accordance with our previous analyses [11], the final TSI in both treatment groups positively correlated with tumor volume at necropsy (R^2^ = 0.89, p = 0.0001; Figure 3D). The mean TSI in the control group significantly increased over the treatment period (32% pre-treatment vs. 57% post-treatment, p = 0.01; Figure 3E), whereas that in the NVP-BEZ235 cohort significantly decreased (36% vs. 20%, p = 0.008; Figure 3F). Every tumor in the control group increased in size; treated tumors all decreased in size. Taken together, these results demonstrate that *in vivo* treatment with NVP-BEZ235 results in significant regression in *PIK3CA* wild-type colonic tumors.

### *In vivo* NVP-BEZ235 treatment of a GEM model for sporadic CRC results in sustained mTORC1 and mTORC2 inhibition, but transient PI3K blockade

**Figure 4:**
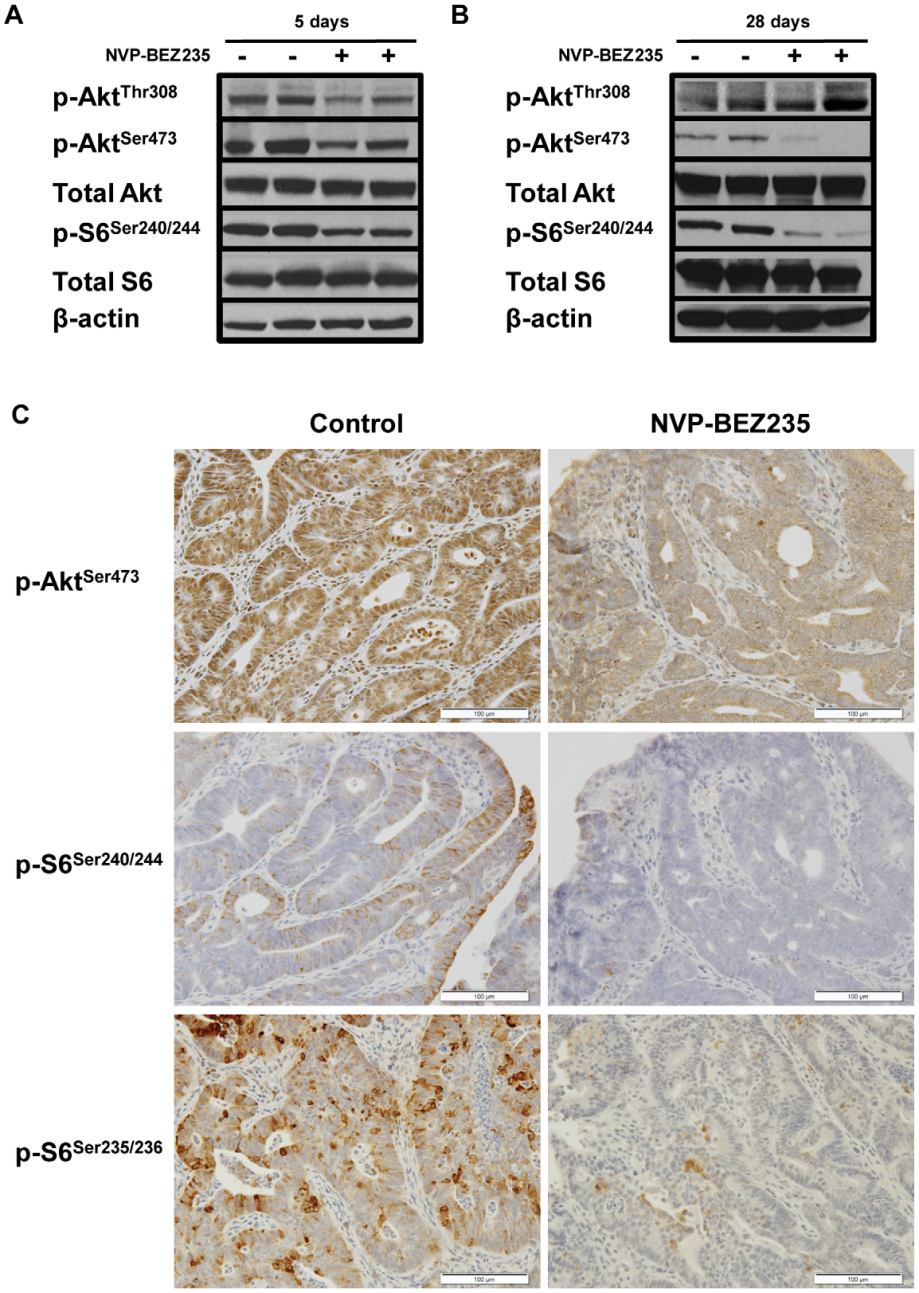
*In vivo* NVP-BEZ235 treatment of a GEM model for sporadic CRC results in transient PI3K blockade inhibition and sustained mTORC1/mTORC2 blockade. Mice with colonic tumors were randomized to treatment with control diluent or 45 mg/kg NVP-BEZ235 by daily oral gavage for five days. Western blot analysis of p-AKT^Thr308^, p-AKT^Ser473^, and p-S6^Ser240/244^ was performed for tumors treated with (−) control diluent or (+) NVP-BEZ235 for (A) five and (B) 28 days. (C) Immunohistochemistry of p-AKT^Ser473^, p-S6^Ser240/244^, and p-S6^Ser235/236^ was performed for tumors treated with control diluent or NVP-BEZ235 for 28 days.

To examine the effects of *in vivo* NVP-BEZ235 treatment on PI3K and mTOR signaling, western blot analysis was performed in colonic tumors that were harvested one hour after final drug dosing on days 5 and 28 of treatment. Western blot analysis for levels of p-AKT^Thr308^, p-S6^Ser240/244^, and p-AKT^Ser473^ was performed as surrogates for activation of the PI3K, mTORC1, and mTORC2 pathways, respectively. A sustained decrease in levels of p-AKT^Ser473^ and p-S6^Ser240/244^ was observed in the tumors of mice treated with NVP-BEZ235, as compared to control diluent (Figure 4A and 4B). These findings were confirmed by tumor immunohistochemistry (Figure 4C). As with our *in vitro* studies, an initial decrease in levels of p-AKT^Thr308^ was observed at five days after treatment, but normalized by 28 days (Figures 4A and 4B). Taken together, these results suggest that *in vivo* NVP-BEZ235 treatment results in sustained inhibition mTORC1 and mTORC2, but transient PI3K blockade.

### *In vivo* NVP-BEZ235 treatment of a GEM model for sporadic CRC inhibits proliferation, has no effect on apoptosis, and blocks tumor angiogenesis

**Figure 5:**
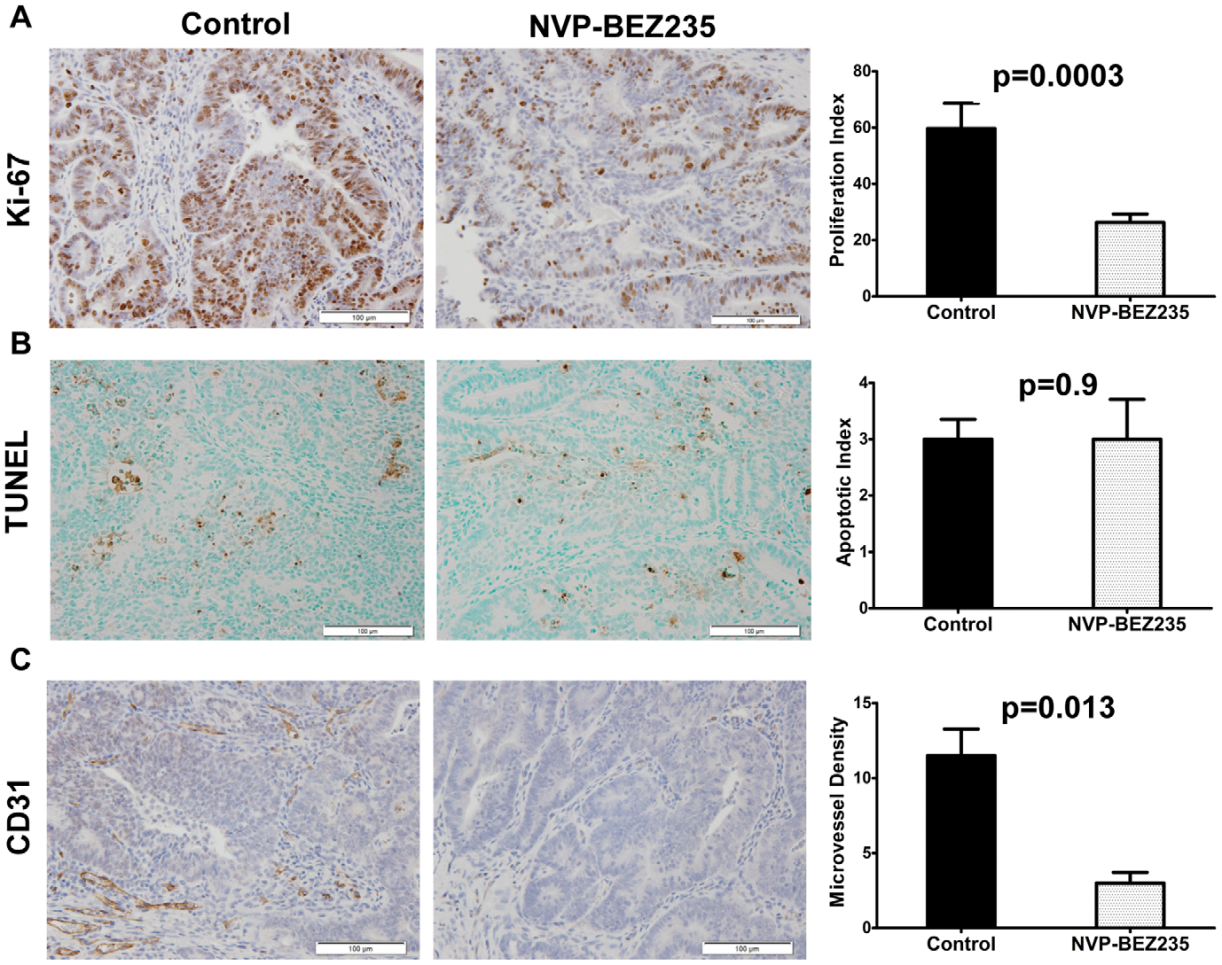
*In vivo* NVP-BEZ235 treatment of a GEM model for sporadic CRC inhibits proliferation, has no effect on apoptosis, and blocks tumor angiogenesis. (A) Representative immunohistochemistry for the proliferation marker KI-67 after treatment with control diluent or NVP-BEZ2355 for 28 days (p = 0.003). KI-67 proliferation index was calculated as average number of positive tumor cells per high power field. (B) TUNEL immunohistochemistry was performed in tumors treated with control diluent or NVP-BEZ235 for 28 days (p = 0.9). TUNEL staining was quantified as percent positive cells per high powered field. (C) Representative immunohistochemistry for the endothelial marker PECAM after treatment with control diluent or 45 mg/kg NVP-BEZ2355 (p = 0.013). Microvessel density was calculated as average number of positive vessels per high power field.

To examine the effects of *in vivo* NVP-BEZ235 treatment on cellular proliferation, colonic tumors were examined by immunohistochemistry for the proliferation marker KI-67. This analysis revealed that proliferation decreased by 56% after 28 days of NVP-BEZ235 treatment (p = 0.003, Figure 5A). To assess the effect of NVP-BEZ235 treatment on cellular apoptosis, colonic tumors were examined by TUNEL assay. No differences were seen between tumors treated with control diluent and NVP-BEZ235 (p = 0.9, Figure 5B). As the PI3K and mTOR pathways play a significant role in tumor angiogenesis [54], [55], we examined the effects of NVP-BEZ235 treatment on microvessel density (MVD) through immunohistochemistry for the endothelial marker CD31. This analysis revealed that MVD decreased by 75% after 28 days of NVP-BEZ235 treatment (p = 0.013, Figure 5C). Taken together, these results suggest that *in vivo* treatment with NVP-BEZ235 results in a significant decrease in tumor proliferation, does not induce cellular apoptosis, and inhibits tumor angiogenesis.

## Discussion

Because of their central role in the initiation and progression of CRC, blockade of the mTOR and PI3K signaling pathways has emerged as a compelling target for the development of novel CRC therapeutics [10], [56]–[59]. We and others have demonstrated the efficacy of mTORC1 pathway inhibition in preclinical CRC models [11], [12]. However, an early clinical trial examining mTORC1 inhibitors in human CRC patients demonstrated modest results, perhaps due to loss of an mTORC1-dependent feedback loop that limits PI3K activation and/or continued mTORC2-mediated activation of AKT [3], [60]. Taken together, it appears dual PI3K/mTOR inhibitors, such as NVP-BEZ235, are required for maximal therapeutic efficacy.

Some studies have suggested that *PIK3CA* mutant cancers are “oncogenically addicted” to PI3K signaling, leading to increased sensitivity to PI3K inhibitor therapy [7], [61], [62]. However, one group has reported no association between *PIK3CA* mutational status and response to PI3K inhibitors [63]. Nonetheless, another group has reported exclusively targeting *PIK3CA* mutant patients for PI3K/mTOR therapy [64]. As *PIK3CA* mutations are seen in only 17% of human CRC, this approach would significantly limit the clinical impact of such agents [53]. Because NVP-BEZ235 equally inhibits the mutant and wild-type forms of *PIK3CA*, we hypothesized that NVP-BEZ235 would have significant efficacy in human wild-type *PIK3CA* CRC [32]. In support of this, we found comparable sensitivity to *in vitro* NVP-BEZ235 treatment in *PIK3CA* mutant (HCT116, kinase domain mutation; DLD-1, helical domain mutation) and *PIK3CA* wild-type (SW480) CRC cell lines, as well as in matched *PIK3CA* mutant and wild-type isogenic cell lines. Direct sequencing of the hot spot regions at exons 9 and 20 of *Pik3ca* in the colonic tumors validated our GEM model as a surrogate for *PIK3CA* wild-type CRC, which we used in our *in vivo* NVP-BEZ235 treatment studies. Taken together, these findings suggest that NVP-BEZ235 treatment may have clinical benefit in the 83% of CRC patients with wild-type *PIK3CA*. To further assess this feature, we sought to examine whether NVP-BEZ235 would be effective in a GEM model for sporadic *PIK3CA* wild-type CRC.

Traditional *in vivo* target validation approaches rely on xenograft platforms. Unfortunately, these models are not truly predictive of response in human patients, because they derive from high passage tumor cell lines grown *in vitro*. These cell lines are implanted into ectopic sites that do not resemble the colonic microenvironment, and therefore fail to recapitulate the heterogeneous nature of cancer and its supporting stroma [65]. Although GEM cancer models address these shortcomings, most GEM models employ germ-line or tissue-wide modification of genes known to be mutated in human CRC. In addition, many CRC GEM models mainly present with small intestinal tumors [66]. To accurately model human sporadic CRC, we have recently described a procedure in which Adeno-Cre is administered to floxed mice to somatically inactivate the *Apc* gene in a stochastic fashion and restrict tumor formation to the distal colon. The reproducible anatomical location of these tumors permits the use of optical colonoscopy to examine individual tumors in a longitudinal fashion [11].

To examine the efficacy of *in vivo* NVP-BEZ235 treatment, we used our GEM model for sporadic CRC. In our studies, there was strong correlation between the final tumor sizes assessed by colonoscopy versus necropsy (R^2^ = 0.89), thus validating our colonoscopy-based tumor sizing protocol. In accordance with our *in vitro* NVP-BEZ235 treatment of human CRC cell lines, colonic tumors from the GEM model decreased by 43% in size after *in vivo* treatment with NVP-BEZ235, whereas control tumors increased in size by 97% (p<0.0001). Similar findings have been reported with another dual PI3K/mTOR inhibitor, PKI-587, in a xenograft model of CRC [67]. Taken together, these results suggest that NVP-BEZ235 would be an effective treatment in human CRC patients.

We studied the effect of NVP-BEZ235 treatment on the PI3K, mTORC1, and mTORC2 signaling pathways. Both *in vitro* and *in vivo* studies demonstrated sustained inhibition of mTORC1 and mTORC2 (reflected in decreased phosphorylation of S6 and AKT^Ser473^, respectively), but transient blockade of PI3K activation by PDK1 (assessed by levels of p-AKT^Thr308^). These findings are consistent with a report demonstrating the parallel roles of mTORC1 and mTORC2 in CRC carcinogenesis [68]. Findings from other cancer models suggest that NVP-BEZ235 prevents mTORC2-dependent AKT^Ser473^ phosphorylation, and that PI3K inhibition with NVP-BEZ235 may be short-lived due to loss of feedback inhibition of PI3K activity by the mTORC1 target S6 kinase [19], [39], [69]. Therefore, we speculate that the addition of a selective PI3K inhibitor with efficacy against wild-type and mutant *PIK3CA* may augment the effectiveness of NVP-BEZ235 therapy. Taken together, our findings suggest that the efficacy of NVP-BEZ235 may derive predominantly from inhibition of mTORC1 and mTORC2 signaling.

We also examined possible mechanisms by which NVP-BEZ235 might induce tumor regression. *In vivo* NVP-BEZ235 treatment of colonic tumors resulted in a 56% decrease in cellular proliferation, but the absence of an induction in apoptosis. Although induction of apoptosis by NVP-BEZ235 treatment has been reported in lung, breast, renal cell carcinoma, and ovarian models [26], [39], [47], the absence of apoptosis has been found after treatment of glioma and sarcoma models [36], [39]. Furthermore, *in vivo* NVP-BEZ235 treatment of colonic tumors resulted in a 75% decrease in microvessel density after NVP-BEZ235 treatment. Similar findings have been described in pancreatic, glioma, and neuroendocrine tumor systems [19], [39], [54]. It is interesting that though we observed a decrease in macroscopic tumor size after *in vivo* NVP-BEZ235 treatment, we did not see a corresponding induction of apoptosis. This finding could be explained by an increase in constitutive cell turnover resulting from a drug-induced inhibition of angiogenesis coupled to a simultaneous drug-induced decrease in cellular proliferation. Because this process occurred slowly over the 28 day time course, frank necrosis would not be seen. Taken together, these findings support the further investigation of combination therapy with NVP-BEZ235, cytotoxic, and/or anti-angiogenic agents in CRC patients.

In summary, we present evidence for the therapeutic efficacy of NVP-BEZ235 in *PIK3CA* wild-type CRC. Furthermore, our results suggest that the efficacy of NVP-BEZ235 treatment may be augmented in if used in combination with additional PI3K inhibition, cytotoxic, and/or anti-angiogenic agents. Taken together, our findings provide compelling evidence for the further exploration of NVP-BEZ235-based therapies in clinical trials for CRC.
